# Supplementary material for: An Emerging Disease of Chickpea, Basal Stem Rot Caused by Diaporthe aspalathi in China
Source: Plants (Basel). 2024 Jul 16;13(14):1950. doi: 10.3390/plants13141950 (PMC11280406; doi:10.3390/plants13141950)
Supplement: Supplementary file 1 [file plants-13-01950-s001.zip › plants-2961843-supplementary.pdf]

Table S1. Information of the *Diaporthe* isolates from diffident hosts used to constructing the phylogenetic tree this study.

Species	Isolate Name	Host	Origin	GenBank Accessions				
				ITS	EF1-a	TUB	CAL	HIS
D. aspalathi	ZD36-1	*Cicer arietinum*	China	MK330963	MK330966	ON323941	ON323944	ON323947
D. aspalathi	ZD36-2	*Cicer arietinum*	China	MK330964	MK330967	ON323942	ON323945	ON323948
D. aspalathi	ZD36-3	*Cicer arietinum*	China	MK330965	MK330968	ON323943	ON323946	ON323949
D. aspalathi	CBS 117168	*Aspalathus linearis*	South Africa	KC343035	KC343761	KC344003	KC343277	KC343519
D. aspalathi	CBS 117169	*Aspalathus linearis*	South Africa	KC343036	KC343762	KC344004	KC343278	KC343520
D. aspalathi	CBS 117500	*Aspalathus linearis*	South Africa	KC343037	KC343763	KC344005	KC343279	KC343521
D. ambigua	CBS 114015	*Pyrus communis*	South Africa	KC343010	KC343736	KC343978	KC343252	KC343494
D. ambigua	CBS 117167	*Aspalathus linearis*	South Africa	KC343011	KC343737	KC343979	KC343253	KC343495
D. ambigua	CBS 187.87	*Helianthus annuus*	Italy	KC343015	KC343741	KC343983	KC343257	KC343499
D. betulae	CFCC 50469	*Betula platyphylla*	China	KT732950	KT733016	KT733020	KT732997	KT732999
D. betulae	CFCC 50470	*Betula platyphylla*	China	KT732951	KT733017	KT733021	KT732998	KT733000
D. biguttulata	CFCC 52584	*Juglans regia*	China	MH121519	MH121561	MH121598	MH121437	MH121477
D. biguttulata	CFCC 52585	*Juglans regia*	China	MH121520	MH121562	MH121599	MH121438	MH121478
D. caulivora	CBS 127268	*Glycine max*	Croatia	KC343045	KC343771	KC344013	KC343287	KC343529
D. caulivora	CBS 178.55	*Glycine soja*	Canada	KC343046	KC343772	KC344014	KC343288	KC343530
D. citri	CBS 134237	*Citrus reticulata*	China	JQ954660	JQ954676	KC357426	KC357465	MF418279
D. citri	CBS 134239	*Citrus sinensis*	USA	KC357553	KC357522	KC357456	KC357488	MF418280
D. eres	CBS 101742	*Fraxinus sp.*	Netherlands	KC343073	KC343799	KC344041	KC343315	KC343557
D. eres	CBS 102.81	*Juglans regia*	Italy	KC343074	KC343800	KC344042	KC343316	KC343558
D. eres	AR5193	*Ulmus sp.*	Germany	KJ210529	KJ210550	KJ420799	KJ434999	KJ420850
D. helianthi	CBS 344.94	*Helianthus annuus*	–	KC343114	KC343840	KC344082	KC343356	KC343598
D. helianthi	CBS 592.81	*Helianthus annuus*	Serbia	KC343115	KC343841	KC344083	KC343357	KC343599
D. infecunda	CBS 133812	*Schinus terebinthifolius*	Brazil	KC343126	KC343852	KC344094	KC343368	KC343610

D. infecunda	LGMF 908	*Schinus terebinthifolius*	Brazil	KC343127	KC343853	KC344095	KC343369	KC343611
D. infecunda	LGMF 912	*Schinus terebinthifolius*	Brazil	KC343128	KC343854	KC344096	KC343370	KC343612
D. longicolla	FAU642	*Glycine max*	USA	KJ590733	KJ590772	KJ610888	KJ612129	KJ659193
D. longicolla	FAU643	*Glycine max*	USA	KJ590731	KJ590770	KJ610886	KJ612127	KJ659191
D. longicolla	FAU657	*Cucumis melo*	USA	KJ590727	KJ590766	KJ610882	KJ612123	KJ659187
D. melonis	CBS 435.87	*Glycine soja*	Indonesia	KC343141	KC343867	KC344109	KC343383	KC343625
D. melonis	CBS 507.78	*Cucumis melo*	USA	KC343142	KC343868	KC344110	KC343384	KC343626
D. novem	CBS 127269	*Glycine max*	Croatia	KC343155	KC343881	KC344123	KC343397	KC343639
D. novem	CBS 127270	*Glycine max*	Croatia	KC343156	KC343882	KC344124	KC343398	KC343640
D. novem	CBS 127271	*Glycine max*	Croatia	KC343157	KC343883	KC344125	KC343399	KC343641
D. novem	CPC 26188	*Citrus japonica*	Italy	MF418426	MF418505	MF418586	MF418260	MF418346
D. novem	CPC 28165	*Citrus aurantiifolia*	Italy	MF418427	MF418506	MF418587	MF418261	MF418347
D. phaseolorum	CBS 113425	*Oleariarani*	New Zealand	KC343174	KC343900	KC344142	KC343416	KC343658
D. phaseolorum	CBS 116019	*Caperonia palustris*	USA	KC343175	KC343901	KC344143	KC343417	KC343659
D. phaseolorum	CBS 116020	*Symphyotrichum subulatum*	USA	KC343176	KC343902	KC344144	KC343418	KC343660
D. sojae	FAU604	*Glycine max*	USA	KJ590716	KJ590759	KJ610872	KJ612113	KJ659205
D. sojae	FAU636	*Glycine max*	USA	KJ590718	KJ590761	KJ610874	KJ612115	KJ659207
D. vaccinii	CBS 118571	*Vaccinium corymbosum*	USA	KC343223	KC343949	KC344191	KC343465	KC343707
D. vaccinii	CBS 122112	*Vaccinium corymbosum*	USA	KC343224	KC343950	KC344192	KC343466	KC343708
D. vaccinii	CBS 122114	*Vaccinium corymbosum*	USA	KC343225	KC343951	KC344193	KC343467	KC343709
D. vaccinii	CBS 135436	*Vaccinium corymbosum*	USA	AF317570	JQ807380	KC843225	KC849457	KJ420877
